# Biology, Pathophysiological Role, and Clinical Implications of Exosomes: A Critical Appraisal

**DOI:** 10.3390/cells8020099

**Published:** 2019-01-29

**Authors:** Arif Tasleem Jan, Safikur Rahman, Shahanavaj Khan, Sheikh Abdullah Tasduq, Inho Choi

**Affiliations:** 1 School of Biosciences and Biotechnology, Baba Ghulam Shah Badshah University, Rajouri 185236, India; 2 Department of Medical Biotechnology, Yeungnam University, Gyeongsan 38541, Korea; shafique2@gmail.com; 3 Department of Bioscience, Shri Ram Group of College (SRGC), Muzaffarnagar 251001, India; khan.shahanavaj@gmail.com; 4 CSIR-Indian Institute of Integrative Medicine, Canal Road, Jammu 180001, India; stabdullah@iiim.ac.in

**Keywords:** exosomes, extracellular transport, diseases, secretory vesicles, stem cells

## Abstract

Exosomes are membrane-enclosed entities of endocytic origin, which are generated during the fusion of multivesicular bodies (MVBs) and plasma membranes. Exosomes are released into the extracellular milieu or body fluids; this process was reported for mesenchymal, epithelial, endothelial, and different immune cells (B-cells and dendritic cells), and was reported to be correlated with normal physiological processes. The compositions and abundances of exosomes depend on their tissue origins and cell types. Exosomes range in size between 30 and 100 nm, and shuttle nucleic acids (DNA, messenger RNAs (mRNAs), microRNAs), proteins, and lipids between donor and target cells. Pathogenic microorganisms also secrete exosomes that modulate the host immune system and influence the fate of infections. Such immune-modulatory effect of exosomes can serve as a diagnostic biomarker of disease. On the other hand, the antigen-presenting and immune-stimulatory properties of exosomes enable them to trigger anti-tumor responses, and exosome release from cancerous cells suggests they contribute to the recruitment and reconstitution of components of tumor microenvironments. Furthermore, their modulation of physiological and pathological processes suggests they contribute to the developmental program, infections, and human diseases. Despite significant advances, our understanding of exosomes is far from complete, particularly regarding our understanding of the molecular mechanisms that subserve exosome formation, cargo packaging, and exosome release in different cellular backgrounds. The present study presents diverse biological aspects of exosomes, and highlights their diagnostic and therapeutic potentials.

## 1. Introduction

The existence of exosomes as extracellular vesicles (EVs) was first reported by Harding et al. and Johnstone et al. [1,2]. Unlike microvesicles (MVs; 100–1000 nm) and apoptotic bodies (50–500 nm), exosomes are membrane-enclosed vesicles of endocytic origin that range in size from 30 to 100 nm. Their secretions to the extracellular milieu and body fluid compartments (bronchoalveolar lavage, synovial fluid, bile, serum, milk, and urine) were reported for a variety of cells (mesenchymal cells, fibroblasts, epithelial cells, platelets, antigen-presenting cells, and tumor cells) [3,4,5,6]. In addition to simple budding at plasma membrane surface (ectosomes, micro-particles, microvesicles), exosomes are produced in a well-organized two-step process that involves membrane invagination and vesicle budding. Exosomes are loaded with different molecules, such as nucleic acids, cytokines, bioactive compounds, and enzymes, and surface-encoded proteins present as receptors on exosomes act on neighboring cells either by inducing signaling pathways or affecting their cellular phenotypes by transferring new genetic material and receptors [7,8,9,10,11]. Furthermore, their secretions to the extracellular milieu influence host immune systems [3,12,13].

Exosome discovery is bearing fruit, as evidenced by an enormous increase in the number of studies on exosome biology and the establishment of scientific societies like the American Society for Exosomes and Microvesicles (ASEM) and the International Society for Extracellular Vesicles (ISEV). This explosive growth of exosome biology even resulted in a journal *Journal of Extracellular Vesicles*, and the establishment of the Exocarta and Vesiclepedia databases, which are dedicated to extracellular vesicles. The extracellular RNA (exRNA) research portal is the result of an initiative by the Extracellular RNA Communication Consortium (ERCC) that provides a catalog of extracellular RNAs (exosomes are potential RNA carriers), and reports on the mechanisms of exRNA generation, secretion, transport, therapeutic uses, and their uses as biomarkers of disease. In addition to their importance as signaling molecules for intercellular communication and regulation, exosomes offer potential means of ameliorating pathogenic immune responses, diagnosing different diseases, and delivering therapeutics. Here, we summarize essential findings of exosome biology, and provide an up-to-date account of their diverse physiological and pathological functions.

## 2. Isolation and Characterization

Exosomes made their entrance into the scientific world three decades ago. Late endosomes were considered as pre-degradative compartments, and the vesicular structures secreted by late lysosomes were considered as the membranous entities of dying cells. As technical problems in the isolation methods hamper their separation from other vesicle types, no consensus was reached on their isolation methods. The existence of exosomes became apparent when reticulocyte culture supernatant was purified by ultracentrifugation, and they were only recognized as functional entities by electron microscopy [14]. Ultracentrifugation at ≥100,000× *g* is routinely used to obtain exosomes from culture supernatants. Although the technique excludes contamination by dead cell debris, it results in mixed fractions of exosomes, protein aggregates, and vesicular structures. Other isolation methods include serial filtration [15], immunoaffinity purification against surface proteins [16], and commercially available kits, which allow rapid, straight forward isolation. Confirmation that isolated vesicles are exosomes is achieved by laser scatter tracking, electron microscopy, and other techniques such as mass spectrometry [17,18,19,20].

Observations of exosomes by whole-mount electron microscopy revealed them to be “saucer-like” or “deflated-football” shaped, believed to be due to vesicle collapse during sample preparation [21]. Although Harding reported in 1983 that exosomes are generated as multivesicular entities (MVEs) [2], their vesicular characteristics were established by Pan and Johnstone in a study of the transition of sheep reticulocytes [22]. The enrichment of Rab GTPases (Rab4 and Rab5), which act as membrane traffic regulators in exosomes, was first reported by Vidal and Stahl [23], and this was followed by a report on major histocompatibility complex class II (MHC-II)-bearing exosomes from B lymphocytes [19] and dendritic cells (DCs) that were capable of stimulating T-cell response [8,24,25]. The presence of Rab11 in exosome secretions and the triggering of exosome secretion by calcium transients were established by Savina et al. [26,27], and Rab 27 and Rab35 were identified as regulatory GTPases by Hsu [28]. Baietti demonstrated the presence of apoptosis-linked gene 2-interacting protein X (Alix), vacuolar protein sorting-associated protein 4 (VPS4), and components of the endosomal sorting complexes required for transport (ESCRT) pathway in exosome secretions [29].

## 3. Exosome Biogenesis

**Figure 1:**
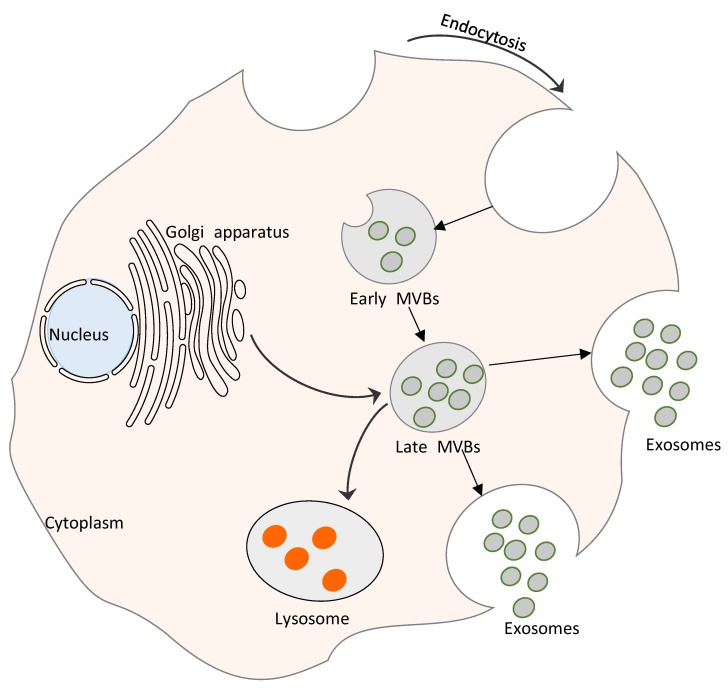
Exosome biogenesis. The process starts with an invagination of the endosomal membrane, and involves Rab GTPase and endosomal sorting complexes required for transport (ESCRTs). The delivery of cargo to recipient cells occurs via ligand–receptor interactions between the exosome and the host cell.

The budding of interluminal vesicles from endosomal compartments and their joining together results in the production of multivesicular bodies (MVBs) [30]. Though some MVBs are destined for lysosome degradation, some fuse with the plasma membrane to cause the release of exosomes into body fluids (in vivo) or to the culture medium (in vitro) [5,31]. Exosome formation involves the participation of specific proteins, especially ESCRTs, which are involved in the sorting of endosomal proteins for loading into MVBs (Figure 1). Furthermore, interactions between ESCRT-I, -II, and -III with mammalian hepatocyte receptor tyrosine kinase substrate (Hrs) and Vps27 sort ubiquitinated cargos, and trigger their transport into the MVB compartment [30,32]. In vitro experiments revealed that ESCRT-I and -II recruitment drives membrane budding and the recruitment of ESCRT-III via Alix, which binds with the tumor susceptibility gene 101 (TSG101) component of ESCRT-I, while ESCRT-I and -II complexes cause the completion of budding [33]. Dissociation of ESCRT from MVB membranes occurs through the involvement of an ATPase, Vps4 [30,32]. Interestingly, similar patterns of exosome formation were observed in dendritic cells (DCs) [6], antigen-presenting cells (APCs) [19], cytotoxic T-lymphocytes (CTLs) [34], Epstein–Barr virus (EBV)-transformed B-cells [19], mastocytes [35], and platelets [36].

## 4. Exosome Composition

**Figure 2:**
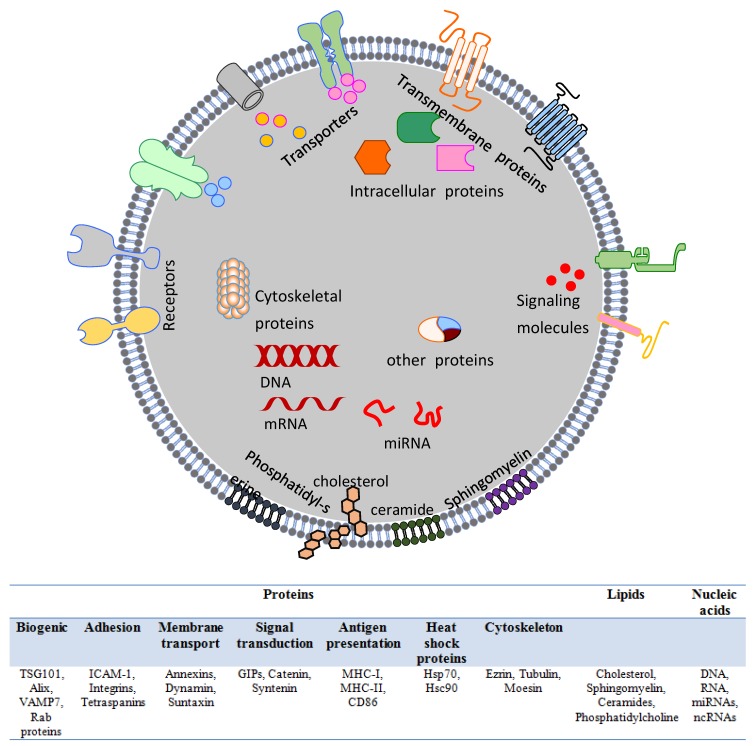
Structure of an exosome. Exosomes exhibit a complex lipid-bilayer surface structure characterized by an array of surface-localized proteins and membranous lipids, which mediate specific targeting and promote cellular uptake.

Fluorescence-activated cell sorting (FACS), Western blotting, and mass spectrometry are commonly employed to decipher the exact compositions and to identify the molecular constituents of exosomes [17,19,37]. Depending largely on their cellular origins, exosomes contain specific sets of protein families of endocytic, cytosolic, and plasma membrane origin. Exosomes are enriched with tetraspanins (cluster of differentiation 9 (CD9), CD26, CD53, CD63, CD81, and CD82), endosome-associated proteins (TSG101, Alix), heat-shock proteins (Hsc70, Hsp90), clathrin, flotillin-1, cytoskeletal elements (ezrin, tubulin, and annexins), Rab proteins, MHC molecules, intercellular adhesion molecule 1 (ICAM-1), co-stimulatory T-cell molecules (CD86), other transmembrane proteins (αM (DCs), α4β1 (reticulocytes)), immunoglobulin A33 (enterocytes), P-selectin (platelets), and matrix metalloproteinases (MMPs) [8] (Figure 2). In addition, lipids, such as ceramides, phosphatidylethanolamine, phosphatidylserine, diacylglyceride, cholesterol, sphingomyelin, and lyso-bisphospatidic acid, were also reported to be present on exosome membranes [38]. Furthermore, exosomes also carry nucleic acid (DNA, messenger RNAs (mRNAs), microRNAs, and other non-coding RNAs) signatures. The levels of different components in exosomes depend largely on the functional states of cells producing them, that is, whether they are stimulated, transformed, rested, or stressed [31].

## 5. Exosome Secretion

Exosome secretion into the extracellular milieu modulates gene expression, function, and even cellular differentiation programs. The protein content and genetic material of exosomes can even change the morphology of a recipient cell by interfering with its signaling components. Though little is known of the mechanisms driving MVB to plasma membrane fusion, a study of reticulocytes revealed that exosome secretion is dependent on vesicular-associated molecular pattern 7 (VAMP7) function [39]. Despite the fact that MVB fusion to the plasma membrane requires vesicular soluble *N*-ethylmaleimide-sensitive factor (NSF) attachment protein receptors (v-SNAREs) and target SNAREs (t-SNAREs), the secretion of exosomes with Wingless (Wnt) as a signature depends on arginine (R)-SNARE Ykt6 [40]. The SNARE complex helps with the energy needed for MVB fusion to the plasma membrane, thereby assisting interaction between the two membranes. Independently of its proton-pumping ability, the V0 subunit of v-ATPase, in association with SNAREs, assists fusion by forming fusion pores [41]. Additionally, Rab proteins (Rab11, Rab27b) form a key component of exosome secretion by facilitating the docking of MVBs to the plasma membrane [42]. Exosome secretion is regulated in part by P2X receptor activation on neutrophils and monocytes and by the lipopolysaccharide (LPS)-induced activations of ATP and Toll-like receptor 4 (TLR4) on dendritic cells [6,42,43].

## 6. Exosome Function

Over the past decade, exosomes were implicated in diverse activities in biological systems, possibly by modulating intercellular communication or action at a distance [21,44]. Their immunomodulatory (immunosuppressive or immune-active) effect is one of the different mechanisms caused by regulation of the deliveries of different constituents to recipient cells [7,45]. These effects attracted the interest of clinical immunologists [21,46]. Because they produce immunosuppressive molecules, exosomes play dual roles in cancer, that is, they can aid the growth and dissemination of cancer cells by overcoming the activities of T-lymphocytes and natural killer cells (NKCs) and/or modulate the immune system by promoting the differentiation of T-regulatory cells or myeloid cells that elicit anti-tumor responses [47,48,49]. In particular, exosomes of dendritic cell origin possess MHC-I and -II on their surface, whose binding to T-cell receptors induce an adaptive immune response by activating CD4^+^ or CD8^+^ T cells [50,51]. However, exosomes from immature DCs reduce adaptive immune responses by inducing apoptosis of the T-cells, thus promoting immunogenic tolerance, as observed in murine models of autoimmune diseases and transplantation [7]. By influencing the balance between pro- and anti-inflammatory effector T-cells, these suppressive exosomes were found to induce the differentiation of T-helper 17 (Th17)/Th1 cells to forkhead box P3 (Foxp3) and Th2 regulatory T cells [52]. Furthermore, Corrado et al. reported on the immune adjuvant potential of exosomes [7]. By acting as antigen-presenting vesicles, exosomes can possibly be exploited to evade graft rejection and treat autoimmune diseases.

Exosomes released by epithelial cells (intestinal epithelial lining) were found to be involved in antigen presentation during inflammatory conditions. It may be that these extracellular vesicles are responsible for providing fixed cells with the ability to act at a distance [53]. In the nervous system, exosomes secreted by cells (neurons, microglia) are utilized in cell communication, and participate in neurite outgrowth formation, neuronal survival, and myelin formation [54]. Moreover, the release of pathogenic proteins (prions, β-amyloid peptides) by exosomes exacerbates central nervous system disorders [55,56,57]. In the liver, exosomes participate in a plethora of processes. Epithelial (hepatocytes, cholangiocytes) exosome production was also reported from stellate and adult liver stem cells [58,59,60,61]. Despite their effects on extracellular signal-regulated kinase (ERK) signaling and on the expression of microRNA 15a (miR-15a), biliary exosomes were found to inhibit the proliferation of cholangiocytes [60,62,63]. Exosomes from mouse hepatocytes contain drug-metabolizing enzymes, such as cytochrome P450 (CYP450) and glutathione *S*-transferase, which are responsible for the detoxification of toxins and drugs in target cells [58,64,65].

Exosomes containing oncogenic materials (oncogenic DNA, their transcripts, and activated oncoproteins) are referred to as oncosomes, and they mediate the intercellular transport of these mutant molecules in a systemic manner [66,67,68,69]. Fibroblast-derived exosomes promote breast cancer cell dynamics via a Wnt signaling pathway [70]. Analysis of the oncosome cargoes circulating in body fluids appear to offer continuous monitoring of the changing molecular make-ups of different cancers. As such, oncosomes offer promising diagnostic tools for specific cancer subtypes and for determining their prevalence and statuses.

## 7. Prospective Applications

Given their wide-ranging functions, exosomes have huge diagnostic and therapeutic potential. By regulating physiological functions, such as angiogenesis, intercellular communication, coagulation, immune response, and cell survival, exosomes are of immense interest to the scientific fraternity worldwide. Their secretions from cells into body fluids (plasma, urine, cerebrospinal fluid (CSF), saliva, and others) are widely reported (Table 1). Exosomes are mini-copies of cells from which they originate [71] in terms of their antigenicity (cancer and immune cells) and their therapeutic potential (stem and antigen primed-cells); thus, exosomes are viewed as being of immense importance for diagnostic and therapeutic applications.

### 7.1. Exosomes in Diagnostics

With specific protein signatures (Alix, TSG101, CD9, CD63, HSP70, and HSP90), RNA (mRNA and miRNA), and characteristic lipid contents, exosomes in body fluids (blood, serum, milk, urine) were investigated as diagnostic markers for the early detection of various diseases [118,119]. Exosomal RNAs from saliva, amniotic fluid, and urine were examined in the context of their use as a diagnostic marker for CD24 polymorphism (C→T; alanine to valine change) associated with a modulation in the progression of multiple sclerosis (MS), chronic hepatitis B, systemic lupus erythematosus (SLE), and giant-cell arthritis [120].

Increases in the miRNA content released as part of exosomes into body fluids provide insight into the progression of the disease [118,121]. Cancer patients exhibit characteristic patterns of RNA and miRNA packaged in circulating MVs, and a major proportion of these are exosomes [122]. Diseases like diabetes, lung cancer, and colorectal cancer have definite miRNA expression patterns [123,124,125,126]. MicroRNA 92a is downregulated in plasma in hepatocellular carcinoma and leukemia [127,128]. Furthermore, serum miRNAs (miR25, miR223) were reported to provide specific miRNA signatures in non-small-cell lung cancer and liposarcoma [129,130]. Serum miR-141 levels were used to differentiate prostate cancer patients and normal controls [131,132].

Human saliva, which is another indispensable source of exosomes, contains nucleic acids and proteins, and provides diagnostic signatures for different diseases [133]. Levels of miRNAs in saliva samples obtained from parotid and submandibular/sublingual regions from healthy controls and patients suffering from Sjogren’s syndrome showed that miRNAs highly expressed in parotid glands were differentially expressed. Michael et al. (2010) reported a marked difference between the six highly expressed miRNAs in Sjogren’s syndrome and healthy controls (Sjogren’s syndrome: hsa-miR-23a, hsa-miR-27b, hsa-miR-29b, hsa-miR-29c, hsa-miR-150, hsa-miR-335; and healthy controls: hsa-let-7c, hsa-miR-17, hsa-miR-128, hsa-miR-150, hsa-miR-212, hsa-miR-1908) [134].

Urine also serves as an efficient source of exosomal markers of urogenital diseases. Proteomic analysis of urine helped identify eight proteins useful for the detection of bladder cancer [135]. Exosomes from urine possess mRNA-encoding protein prostate cancer-associated 3 (PCA3) and the transmembrane protease serine 2 (TMPRSS2)–erythroblast transformation-specific (ETS)-related gene (ERG) fusion product, an entity over-expressed in prostate cancer [136]. The detection of proteins, mRNAs, and miRNAs in patient serum, saliva, and/or urine offers a unique means of diagnosing and detecting early disease. Exosomes provide more cost-effective, accurate, and non-invasive diagnostic tools than traditional invasive methods, and potentially better outcomes [137]. By acting as antigen-presenting vesicles, exosomes derived from malignant effusions, neoplastic cells, or tumor-pulsed dendritic cells are currently being explored for use as non-invasive biomarkers in the diagnosis of cancer, in addition to their use as a prognostic marker in cardiovascular and neurodegenerative disorders (Alzheimer’s and Parkinson’s) and for the management of infectious diseases (diphtheria, tuberculosis), autoimmune diseases (lupus erythromatosus and rheumatoid arthritis). Accordingly, pharmaceutical companies, such as Exosomics and Exosome Diagnostics, are focusing on the commercialization of exosome-based diagnostics.

### 7.2. Exosomes in Therapeutics

The properties of exosomes, which include bioavailability, distribution, and stability under in vivo and in vitro conditions, and their abilities to cross the blood–brain barrier (BBB) and regulate gene expression via the transfer of miRNA and small interfering RNA (siRNA) to target cells means they are preferred over other EVs as potential therapeutics [137]. Furthermore, because exosomes encapsulate and, thus, protect contents from degradation, their use in disease immunotherapy is viewed with considerable optimism. The successful completion of two independent phase I trials of dexosomes (autologous DC-derived exosomes) for treating non-small-cell lung (NSCL) cancer confirmed their potential therapeutic applications [15,24,138,139]. Because they topically present MHC-I, MHC-II, and CD1, dexosomes induce innate and adaptive immune responses. More specifically, they activate cytotoxic T-cells, induce tumor rejection in mice, and promote NKC activation and NKC-dependent anti-tumor effects in immunocompetent mice [140]. Furthermore, dexosome immunotherapy is considered safe [141].

Mesenchymal stem cells (MSCs) are stromal cells with well-known therapeutic potentials. These cells are present in bone marrow, umbilical cord, and adipose tissue [142]. The exosomes derived from MSCs added a new paradigm of therapeutic applications in regenerative medicine. MSC-Ex therapies are preferred over MSC transplantation because they induce fewer immune responses, increase safety, and reduce storage, shipment, and administration concerns. Thus, “off-the-shelf” products with negligible immunogenicity are readily developed using exosomes. By using MSC exosomes rather than MSCs, limitations regarding replicating cell transplantation and safety concerns are much alleviated. The administration of MSC-derived MVs intravenously was found to accelerate recovery after glycerol-induced acute renal injury in SCID mice [143], which argues well for the use of MSC-Ex as an adjuvant therapy for acute renal injury. Yan et al. (2017) reported the use of human umbilical cord MSC-derived exosomes (hucMSC-Ex) for the treatment of liver disease, and assessed their efficacy and their action mechanism [144]. On measuring antioxidant activities and assessing the hepato-protective effect of hucMSC-Ex in vitro and in vivo, these workers found that hucMSC-Ex (at a systemic dose of 16 mg/kg) reduced reactive oxygen species (ROS) and malondialdehyde (MDA) levels and, thus, increased the viabilities of L02 cells exposed to CCl_4_ or H_2_O_2_ and effectively rescued recipient mice from CCl_4_-induced liver failure. The mechanism of rescue by hucMSC-Ex-derived cells was attributed to glutathione peroxidase 1 (GPX1), which detoxifies CCl_4_ and H_2_O_2_, thereby reducing oxidative stress and apoptosis. It was also shown that a knockdown of GPX1 in hucMSCs abolished the antioxidant and anti-apoptotic abilities of hucMSC-Ex, and reduced the hepato-protective effects of hucMSC-Ex in vitro and in vivo.

By aiding the adhesion of hematopoietic stem-cell progenitor cells to endothelia, microvesicles derived from platelets (PMVs) supported the engraftment of transplanted stem cells in lethally irradiated mice [145]. Beltrami et al. (2017) observed enrichment of miRNAs of potential cardiovascular origin in the exosomes of pericardial fluid [146]. Pericardial fluid (PF) is an ultrafiltrate of plasma found in the pericardium. On the mechanistic level, they demonstrated the proangiogenic role of miRNA let-7b-5p along with its inhibitory effect on *TGFBR1* in ECs, following delivery of functional let-7b-5p via PF exosomes. Downregulation of let-7b-5p miRNA in PF exosomes impaired the angiogenic response by ECs. At a functional level, the authors reported that PF exosomes enhanced survival, proliferation, and networking of cultured endothelial cells (ECs), and restored the pro-angiogenic function of ECs depleted of their endogenous miRNA content.

Cardiosphere-derived cells (CDCs) induce the therapeutic regeneration of the infarcted human heart by stimulating angiogenesis and causing functional improvements of infarcted myocardium. Interestingly, CDCs reduced scar sizes and the growth of new functional myocardium, which was previously considered an irreparable form of injury [147]. Ibrahim et al. (2014) showed CDC exosomes enhanced angiogenesis and promoted cardiomyocyte survival and proliferation [148]. The authors demonstrated the effects of CDC exosomes on angiogenesis, cardiomyocyte proliferation, and apoptosis using human umbilical cord endothelial cells (HUVECs), and found CDC exosomes stimulated angiogenesis, improved cardiac function, and increased viable mass after myocardial infarction (MI) in an established preclinical model versus normal human dermal fibroblast (NHDF) exosomes. A comparative study of CDC- and NHDF-derived exosomes showed CDC exosomes promoted tube formation by HUVECs, which is indicative of enhanced angiogenesis. The authors compared the miRNA repertoires of CDC and NHDF exosomes using a PCR array and showed that CDC exosomes are rich in miR-146a, which is a main mediator of the beneficial effects of these exosomes against MI. In addition, they showed inhibition of exosome secretion using GW4869 (a reversible inhibitor of neutral sphingomyelinase), which prevents exosome release and attenuates CDC benefits by inhibiting exosome secretion.

Exosomes are known to have neuroprotective effects, that is, they aid neuron healing and the regeneration of peripheral nerves, and also act as mediators of neurodegenerative diseases. Furthermore, their ability to cross the blood–brain barrier makes them indispensable neurotherapeutic carriers of drugs and therapeutics. In the nervous system, exosome-mediated neuronal communication facilitates cell-to-cell interactions. The transfer of miRNAs and protein entities from glial cells to neural exons was reported a few years ago [149]. Sulfatides, galactocerebrosides, and cholesterol are myelin lipids primarily found in myelin sheaths and they are required for nerve conduction; they showed their release into the exosomes via oligodendrocytes [150]. The involvement of MVBs in Alzheimer’s patients was first suspected when it was observed that more MVBs were present in forebrain cortical neurons, and in Huntington’s disease, mutated huntingtin protein was found to accumulate in MVBs.

## 8. Conclusions

Exosomes are importantly involved in intercellular communication and in the pathogeneses of various human diseases. Because exosomes are readily accessible in body fluids, their genetic profiles provide new diagnostic and prognostic tools and open new therapeutic possibilities. Their abilities to carry mRNAs, miRNAs, and non-coding RNAs provide an efficient means of controlling protein expressions at a distance in different target cells. The use of exosomes as delivery vehicles offers significant advantages over existing delivery systems due to their small size, non-toxic natures, and target specificities [151]. Interest in exosomes by scientists and physicians increased markedly, but we are still in an early stage of deciphering the molecular mechanisms involved in exosome biogenesis and cargo recruitment.

As our understanding of the biology of exosomes increases, so will our knowledge of design principles and exosomal conjugates. DC-derived exosomes engineered to express rabies virus glycoprotein showed positive results in the delivery of siRNA across the BBB in murine models [152]. To facilitate the release of therapeutic cargoes under specific conditions, bioengineered liposomes and polymer nano-carriers based on exosome templates are needed to achieve better targeting and increase drug uptakes [153,154]. The use of lipid–protein compositions that increase exosome fusion to recipient cells and the engineering of liposomes for exosomic proteins like tetraspanins hold great promise for the targeted delivering of drugs to tumor tissues [155,156,157]. Although exosomes provide a new platform for therapy and biomarker development, many challenges remain to be overcome, but continued progress in this field is sure to reveal the secrets responsible for their physiological and pathological roles.

## Figures and Tables

**Table 1:** List of the top exosome proteins. The classification is based on numbers of reported occasions, and includes protein names, symbols, report times, cell origins and species, and methods used for identification. (Reported species: R = rat, M = mouse, H = human, B = bovine, D = *Drosophila*).

No.	Proteins (Species Found)	Symbol	Tissue/Cell/Body Fluids	Identification Methods	References
1.	Programmed cell death 6 interacting protein (R, M, H)	PDCD6IP	Pancreatic, colorectal, breast, ovarian, liver, and brain cancer cells, hepatocytes, neural stem cells, embryonic fibroblast, dendritic cells, platelets, macrophages, reticulocytes, urine, serum, saliva	Mass spectrometry, Western blotting	[72,73,74,75,76,77,78,79,80]
2.	Heat-shock protein 8 (R, M, H, B)	HSPA8	Prostate, colorectal, and brain cancer cells, macrophages, mast cells, adipocytes, reticulocytes, platelets, urine, milk, serum, saliva	Mass spectrometry, Western blotting, RNA sequencing	[70,77,81,82,83,84,85,86,87,88]
3.	Annexin A2 (R, M, H, B)	ANXA2	Ovarian, colorectal, and breast cancer cells, microglia, dendritic cells, macrophages, fibroblasts, hepatocytes, adipocytes, reticulocytes, platelets, thymus, urine, milk, saliva	Mass spectrometry, Western blotting, RNA sequencing	[70,77,80,81,83,84,86,87,89,90,91,92]
4.	Syndecan-binding protein (R, M, H, B)	SDCBP	Pancreatic, ovarian, colorectal, prostate, and brain cancer cells, dendritic cells, macrophages, mast cells, reticulocytes, hepatocytes, platelets, fibroblasts, urine, milk, saliva	Mass spectrometry, Western blotting	[24,77,82,83,84,85,87,93]
5.	Heat-shock protein 90 alpha class A member 1 (R, M, H, B)	HSP90AA1	Ovarian, colorectal, prostate, and bladder cancer cells, neural stem cells, macrophages, mast cells, adipocytes, reticulocytes, hepatocytes, pancreatic cells, platelets, fibroblasts, urine, milk, serum, saliva	Mass spectrometry, Western blotting, microarray	[70,77,80,81,83,84,86,94,95,96,97,98]
6.	Tumor susceptibility gene 101 (R, M, H, B, D)	TSG101	Colorectal, liver, prostate, and bladder cancer cells, neural stem cells, dendritic cells, macrophages, reticulocytes, hepatocytes, platelets, urine, milk, *Drosophila* s2 cells.	Mass spectrometry, Western blotting	[24,58,76,77,80,83,87,97,99,100,101,102]
7.	Eukaryotic translation elongation factor 1 alpha 1 (R, M, H, B)	EEF1A1	Ovarian, colorectal, prostate, and bladder cancer cells, mast cells, adipocytes, hepatocytes, reticulocytes, dendritic cells, platelets, macrophages, pancreatic cells, fibroblasts, thymus, urine, saliva, milk	Mass spectrometry, RNA sequencing, microarray	[24,58,70,77,80,81,83,84,86,87,93,94,103]
8.	Tyrosine-3-monooxygenase/ tryptophan-5-monooxygenase activation protein, zeta (R, M, H, B)	YWHAZ	Ovarian, colorectal, prostate, and bladder cancer cells, mast cells, hepatocytes, reticulocytes, dendritic cells, platelets, pancreatic cells, fibroblasts, thymus, urine, saliva, milk	Mass spectrometry, microarray	[24,70,77,83,84,94,95,104,105,106]
9.	Eukaryotic translation elongation factor 2 (R, M, H, B)	EEF2	Ovarian, pancreatic, bladder, colorectal, prostate, and breast cancer cells, adipocytes, hepatocytes, reticulocytes, platelets, thymus, saliva, urine, milk	Mass spectrometry, RNA sequencing, microarray	[58,70,77,80,81,83,85,86,87,93,94,95,98,103,106,107,108,109,110,111]
10.	Heat-shock protein 90 alpha class B member 1 (R, M, H, B)	HSP90AB1	Ovarian, pancreatic, bladder, colorectal, and prostate cancer cells, dendritic cells, macrophages, mast cells, neural stem, cells, pancreatic cells, adipocytes, hepatocytes, reticulocytes, platelets, thymus, urine, milk, serum	Mass spectrometry, Western blotting,	[24,58,77,79,80,81,83,85,86,88,94,95,96,98,103,106,109,111]
11.	Annexin 5 (R, M, H)	ANXA5	Ovarian, bladder, colorectal, and prostate cancer cells, adipocytes, hepatocytes, reticulocytes, platelets, thymus, urine, serum, dendritic cells, fibroblast, macrophages, mast cells, pancreatic cells, urine	Mass spectrometry, Western blotting, fluorescence-activated cell sorying	[24,58,70,73,77,80,81,85,87,88,94,95,103,109,110,111,112,113]
12.	Fatty-acid synthase (R, M, H, B)	FASN	Adipocytes, hepatocytes, reticulocytes, fibroblast, pancreatic cells, milk, breast milk, bladder, colorectal, ovarian, and prostrate cancer cells, platelets, serum, thymus, urine	Mass spectroscopy, Western blotting, RNA sequencing	[58,70,73,77,81,83,85,87,93,95,103,106,109,111,114,115]
13.	Tyrosine-3-monooxygenase/ tryptophan-5-monooxygenase activation protein, epsilon (R, M, H, B)	YWHAE	Adipocytes, hepatocytes, reticulocytes, urine, milk, mast cells, pancreatic cells, bladder, colorectal, ovarian, and prostrate cancer cells, platelets, saliva, thymus, urine	Mass spectroscopy	[58,73,77,81,83,85,88,94,95,103,106,109,110,111,113]
14.	Clathrin heavy chain (Hc) (R, M, H)	CLTC	Adipocytes, hepatocytes, reticulocytes, fibroblast, macrophages, bladder, colorectal, ovarian and prostate cancer cells, plasma, platelets, saliva, thymus, urine	Mass Spectroscopy, RNA Sequencing	[58,70,73,77,80,81,85,87,103,106,109,110,111,116,117]
